# High-Temperature Oxidation Behavior of AlTiNiCuCo_x_ High-Entropy Alloys

**DOI:** 10.3390/ma14185319

**Published:** 2021-09-15

**Authors:** Junfeng Wang, Qiaobai He, Guanqi Liu, Qi Zhang, Guotan Liu, Zhihao Huang, Xiaoshuo Zhu, Yudong Fu

**Affiliations:** 1 Department of Material Science and Chemical Engineering, Harbin Engineering University, Harbin 150001, China; sjtu-wangjunfeng@sjtu.edu.cn (J.W.); heqiaobai@hrbeu.edu.cn (Q.H.); liuguanqi@cclxgroup.com (G.L.); liuguotan097@163.com (G.L.); hithzh@gmail.com (Z.H.); 2 Shanghai Key Lab of Advanced High-Temperature Materials, Precision Forming and State Key Lab of Metal Matrix Composites, School of Materials Science and Engineering, Shanghai Jiao Tong University, Shanghai 200240, China; 3 Chengdu Aircraft Industrial (Group) Co., Ltd., Chengdu 610072, China; rbclc@avic.com; 4 Department of Mechanical Engineering, Xinjiang University, Urumqi 830046, China; zhu-xiaoshuo@hrbeu.edu.cn

**Keywords:** high-entropy alloy (HEA), high-temperature oxidation resistance, oxidation kinetics, microstructure morphology, phase composition and oxidation mechanism

## Abstract

In this study, the high-temperature oxidation behavior of a series of AlTiNiCuCo_x_ high-entropy alloys (HEAs) was explored. The AlTiNiCuCo_x_ (x = 0.5, 0.75, 1.0, 1.25, 1.5) series HEAs were prepared using a vacuum induction melting furnace, in which three kinds of AlTiNiCuCo_x_ (x = 0.5, 1.0, 1.5) alloys with different Co contents were oxidized at 800 °C for 100 h, and their oxidation kinetic curves were determined. The microstructure, morphology, structure, and phase composition of the oxide film surface and cross-sectional layers of AlTiNiCuCo_x_ series HEAs were analyzed using scanning electron microscopy (SEM), energy-dispersive spectrometry (EDS), and X-ray diffraction (XRD). The influence of Co content on the high-temperature oxidation resistance of the HEAs was discussed, and the oxidation mechanism was summarized. The results indicate that, at 800 °C, the AlTiNiCuCo_x_ (x = 0.5, 1.0, 1.5) series HEAs had dense oxide films and certain high-temperature oxidation resistance. With increasing Co content, the high-temperature oxidation resistance of the alloys also increased. With increasing time at high temperature, there was a significant increase in the contents of oxide species and Ti on the oxide film surface. In the process of high-temperature oxidation of AlTiNiCuCo_x_ series HEAs, the interfacial reaction, in which metal elements and oxygen in the alloy form ions through direct contact reaction, initially dominated, then the diffusion process gradually became the dominant oxidation factor as ions diffused and were transported in the oxide film.

## 1. Introduction

In 2004, Yeh et al. [1,2,3] broke away from the design concept of common alloy materials and proposed melting alloys of multiple elements with high mixed entropy, which were called “multi-principal-element high-disorder alloys” and were later referred to as “high-entropy alloys (HEAs)”. HEAs have excellent properties, such as high hardness, high wear resistance, and high corrosion resistance due to their unique phase composition and structure [4,5,6,7,8,9,10].

In recent years, high-temperature oxidation behavior has become the basic performance index and an important basis for judging the service performance and service life of an alloy, and the high-temperature oxidation performance of HEAs has also received much attention [11]. Y. Garip et al. [12] studied the high-temperature oxidation resistance of the CoCrFeNiAl_x_ series HEAs. The results indicated that the oxidation rates of all the oxidized HEAs increase with either increasing temperature or decreasing Al content, and the best oxidation resistance at temperatures of 800, 875, and 950 °C was observed with CoCrFeNiAl HEA. The oxidation behavior of AlCoCrFeNi HEA at 1100 °C was investigated by Jie Lu et al. [13] in 2021. They found that this HEA exhibits an extremely low oxidation rate and shows good resistance to oxide scale spallation. Butler and Liu [14,15] and others studied the oxidation behavior of Al_x_CrCoNiFe(Si) at high temperature. It was found that when the Al content (atomic fraction) is 10%, the oxidation kinetics of the alloy at 1050 °C conform to parabolic law [16,17], and the oxidation resistance at high temperature is the best among the various Al contents that were tested.

However, the research on high-temperature oxidation of HEAs is still limited, and many issues in the process of high-temperature oxidation are not very clear due to the complexity of alloy compositions [18]. On the basis of previous studies, our group proposed a new high-entropy alloy with a new component and performed first-principles calculations [19] on its related properties. Subsequently, we carried out research on the high-temperature oxidation properties of this new high-entropy alloy. In this paper, high-temperature oxidation experiments on three kinds of AlTiNiCuCo_x_ (x = 0.5, 1.0, 1.5) series HEAs were carried out to investigate the high-temperature oxidation resistance of the HEAs under the same temperature. The oxidation kinetics curve was derived from the experimental results. The phase composition, microstructure, and structure of the oxide layer were analyzed by X-ray diffraction (XRD), scanning electron microscopy (SEM), and energy-dispersive spectrometry (EDS), and the oxidation mechanism was discussed. This paper can provide a reference for the design of new HEAs with high-temperature oxidation resistance.

## 2. Experimental Procedure

**Figure 1:**
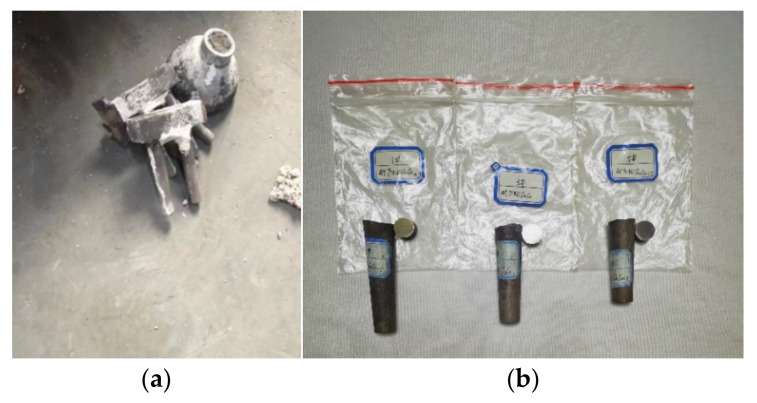
(**a**) High-entropy alloy ingot, and (**b**) polished samples.

Based on the different physical and chemical properties of each element, quinary alloys of Al, Ti, Ni, Cu, and Co with different proportions of Co (x = 0.5, 0.75, 1.0, 1.25, 1.5) were prepared. The purity of each of the five metal element materials was above 99.5%. AlTiNiCuCo_X_ HEAs were prepared by smelting in a model JB-VPC-50 semi-continuous vacuum induction melting furnace (Baofengshoushi Equipment Co., Ltd., Shenzhen, China). Cylindrical ingots of AlTiNiCuCo_X_ HEA with Co contents of 0.5, 1.0, and 1.5 were cut into small pieces, and the surface of each sample was polished with sandpaper, for which 1000 grit was used for the finishing (Figure 1). The small pieces were grouped into an SXL-1008 program-controlled box resistance furnace for the oxidation experiments, and the oxidation temperature was set at 800 °C. The crucibles containing three components were taken out at 25, 50, 75, and 100 h. Pieces were cooled to room temperature and then weighed on an electronic balance with a precision of 0.0001 g. The pieces were measured several times in parallel and the data were recorded; then, the oxidation weight increase G was calculated, and the oxidation kinetics curve was drawn.

The AlTiNiCuCo_x_ series HEAs were further ground to 2000 gritsandpaper fineness and then polished, cleaned, and blow dried. The high-entropy alloy phases were analyzed using X-ray diffraction (XRD) (PANAlytical B.V., Almelo, The Netherlands) with a scanning rate of 4° per minute, an angle range from 20° to 110°, a Cu target, and a wavelength of 1.54 nm. Phase changes in the high-entropy alloy series with different Co content were analyzed according to the processed and plotted test data.

The AlTiNiCuCo_x_ series HEAs were further ground to 3000 grit sandpaper fineness. We polished the surface of the sample until no scratches could be seen under an optical microscope. Then, we created a pit on the polished surface of the sample via corrosion, whereby diluted aqua regia was applied twice for about 60 s each time, and the surface was then cleaned with ethanol and blow dried. The morphology of the oxidized layer on the surface of the oxidized sample was observed using a scanning electron microscope (SEM) (JEOL, Beijing, China). An energy-dispersive spectrum (EDS) analyzer (JEOL, Beijing, China) attached to the scanning electron microscope was used to analyze the composition and distribution of the elements in different areas of the sample surface. The points with special morphologies and different areas were comprehensively analyzed through point scanning, line scanning, and surface scanning.

## 3. Results and Discussion

### 3.1. Oxidation Behavior of AlTiNiCuCo_x_ Series High-Entropy Alloys at 800 °C

The relationship between the oxidation weight per unit area and time at high temperature can be expressed using the following equation:

(1) $$\Delta m=K_{1}t^{n}$$

where ∆*m* is the weight gain per unit surface area, *K*_1_ is the rate constant, *t* is time, and *n* is the time index.

In terms of the oxidation reaction of a metal alloy, there are two extreme cases [20,21,22]. The first occurs when oxygen is in direct contact with the surface. Upon contact, the rate control mechanism of the oxidation reaction is a gas–metal interface reaction. Under this condition, the mass gain per unit surface area increases linearly with oxidation time, and the time index is *n* = 1. The second extreme case occurs when a crack-free stable surface oxide film is formed, and the alloy does not directly make contact with gaseous oxygen. Under this condition, oxidation layer diffusion becomes the rate-controlling process of the oxidation reaction. In this case, the oxidation rate decreases with time (*n* = 0.5).

**Figure 2:**
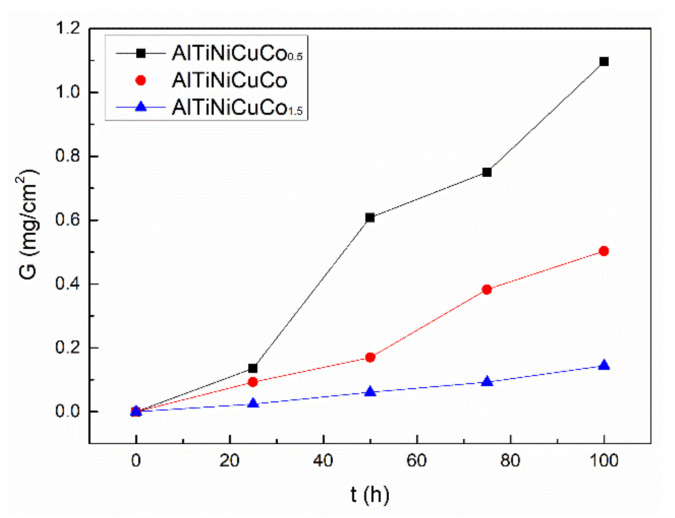
Kinetic curve of AlTiNiCuCo_x_ series HEAs after oxidation at 800 °C.

Table 1 shows the oxidation weight gain per unit area of AlTiNiCuCo_x_ (x = 0.5, 1.0, 1.5) series HEAs at different oxidation times. Through comparison, it was found that after oxidation for 100 h, the weight gain per unit area of the AlTiNiCuCo_0.5_ alloy was 1.096 mg/cm^2^, compared with 0.503 and 0.144 mg/cm^2^ for the AlTiNiCuCo_1.0_ and AlTiNiCuCo_1.5_ alloys, respectively. Therefore, for the same oxidation time, the oxidation weight per unit area decreased with increasing Co content in AlTiNiCuCo_x_ series HEAs. The oxidation kinetic curve of AlTiNiCuCo_x_ (x = 0.5, 1.0, 1.5) series HEAs is shown in Figure 2. It can be seen that at 800 °C, the oxidation weight gain trends of the three alloys were similar. The oxidation weight gain gradually increased with the extension of oxidation time, and the process mainly belonged to the first oxidation case. The average oxidation rates, as obtained by fitting straight lines to the three kinetic curves, were 0.012 mg/(cm^2.^h) (AlTiNiCuCo_0.5_), 0.006 mg/(cm^2.^h) (AlTiNiCuCo_1.0_), and 0.002 mg/(cm^2.^h) (AlTiNiCuCo_1.5_). This indicated that the oxidation resistance of the AlTiNiCuCo_x_ series HEAs at high temperature was closely related to their Co content.

### 3.2. XRD Analysis of The Oxide Layer

**Figure 3:**
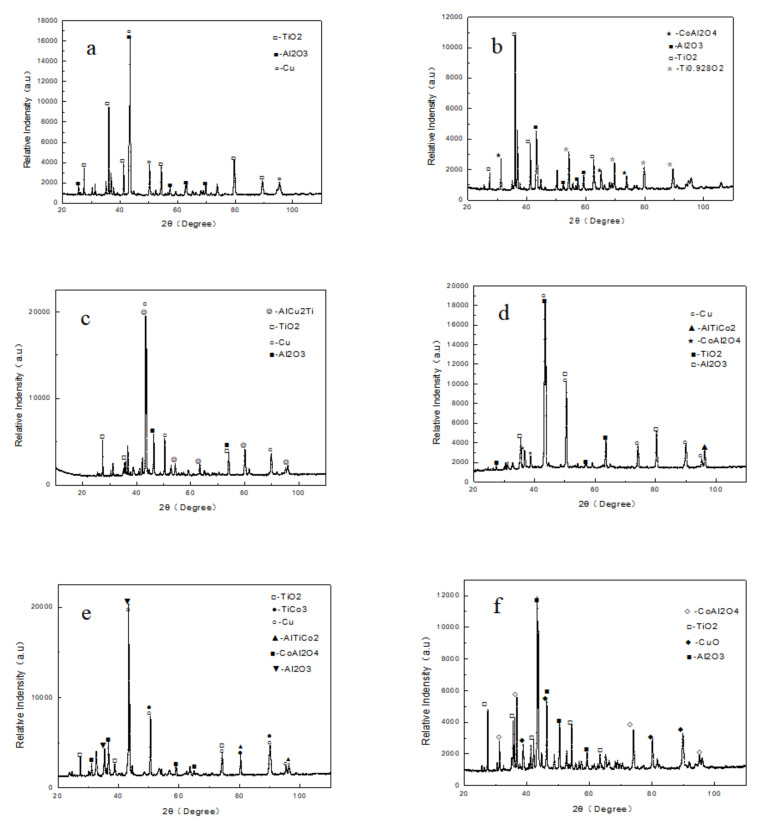
XRD patterns of AlTiNiCuCo_x_ series HEAs after oxidation for different times at 800 °C: (**a**) AlTiNiCuCo_0.5_ oxidized for 50 h; (**b**) AlTiNiCuCo_0.5_ oxidized for 100 h; (**c**) AlTiNiCuCo_1.0_ oxidized for 50 h; (**d**) AlTiNiCuCo_1.0_ oxidized for 100 h; (**e**) AlTiNiCuCo_1.5_ oxidized for 50 h; (**f**) AlTiNiCuCo_1.5_ oxidized for 100 h.

In order to investigate the oxidation products of AlTiNiCuCo_x_ (x = 0.5, 1.0, 1.5) series HEAs at 800 °C, the three alloys were analyzed using X-ray diffraction. Figure 3 presents the XRD patterns of the AlTiNiCuCo_x_ series HEAs for different oxidation times with respective Co contents of 0.5, 1.0, and 1.5.

In XRD analysis of the oxide film surfaces of the AlTiNiCuCo_x_ series HEAs after oxidation for 50 and 100 h at 800 °C, these three alloys were found to all be composed of BCC and FCC phases, and there were diffraction peaks of the matrix phase in the oxide films of all three HEAs after oxidation for 50 h. These indicate that the oxide films were thin at this time. However, after oxidation for 100 h, there were diffraction peaks of the matrix phase only in the alloy with a Co content of 1. Only the diffraction peak of the oxide existed in the other two alloys within the accuracy of the XRD test. Therefore, when the Co content was 1, the oxide film of the HEA was thinner. All three HEAs oxidized for 100 h at 800 °C formed oxides, namely TiO_2_, Al_2_O_3_, and CoAlO_4_.

### 3.3. Surface Morphology of The Oxide Film

**Figure 4:**
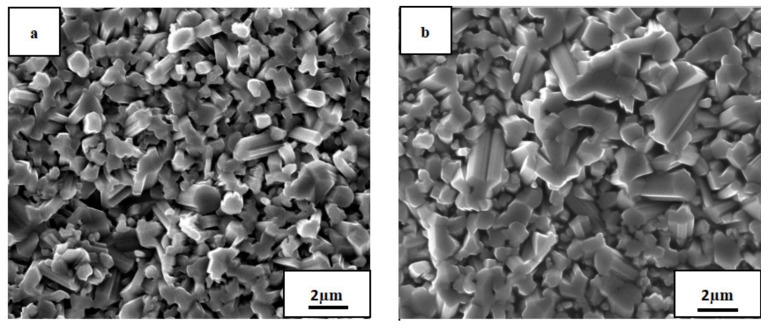
SEM micrographs of AlTiNiCuCo_0.5_ alloy after oxidation for (**a**) 50 h and (**b**) 100 h.

Figure 4 presents SEM micrographs of the oxide film surface of the AlTiNiCuCo_0.5_ alloy oxidized at 800 °C for 50 and 100 h. Table 2 and Table 3 present EDS analysis results of the corresponding regions in the two SEM images.

In Figure 4, it is clear that the oxide film after 50 h oxidation had a more uniform and compact structure and smaller crystal grain size. However, after oxidation for 100 h, the crystal grain size became bigger, and the crystal grain size uniformity was worse.

According to the EDS analysis, the contents of Ti, Al, and O were relatively high after oxidation at 800 °C for 50 h. From this, combined with the results of XRD analysis of the AlTiNiCuCo_0.5_ alloy oxidation film surface after oxidation at 800 °C for 50 h, it can be concluded that TiO_2_ and Al_2_O_3_ were formed. When the oxidation time was 100 h, according to the XRD analysis on the surface of the oxide layer, a new oxide, CoAl_2_O_4_, was also generated. The content of Ti also increased for 100 h oxidation samples. Combined with the results of XRD analysis, this indicates that, in addition to TiO_2_, Ti and O also generated the compound Ti_0.928_O_2_. This indicates that with increasing oxidation time, the overall oxide content increases.

**Figure 5:**
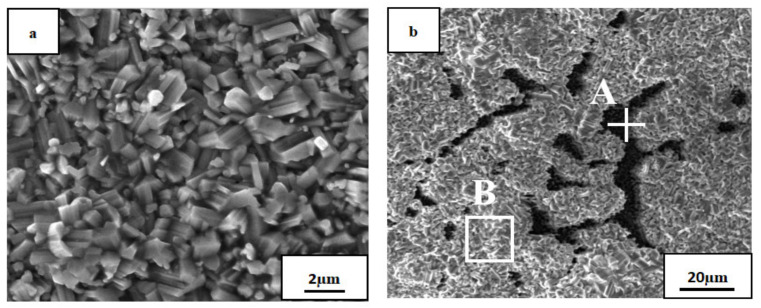
SEM micrographs of AlTiNiCuCo_1.0_ alloy after oxidation for (**a**) 50 h and (**b**) 100 h. A and B are selected areas for EDS analysis.

Figure 5 presents SEM micrographs of the oxide film of the AlTiNiCuCo_1.0_ alloy oxidized at 800 °C for 50 and 100 h, and Table 4 and Table 5 present EDS analysis results of the corresponding regions in the two SEM images.

It can be seen that after 50 h oxidation, the microstructure had the same characteristics of fine grains, uniformity, and compactness as that with 0.5 Co content. After oxidation for 100 h, the microstructure obviously consisted of two parts: a black area and a large gray area.

From the results of EDS energy spectrum analysis, it can be seen that the Ti, Al, and O contents were still high (Table 4 and Table 5). From the XRD analysis of the oxide film above, it can be concluded that two oxides, TiO_2_ and Al_2_O_3_, were formed in the oxide film. However, compared with the AlTiNiCuCo_0.5_ alloy oxidized for 50 h, the EDS analysis showed significantly increased Cu content in AlTiNiCuCo_1.0_, which indicates that the oxide film was thinner due to Cu in the substrate during EDS analysis. Comparing Parts A and B in Table 5 and combining the results with those from XRD analysis, we can infer that there were two oxides, TiO_2_ and Al_2_O_3_, at A, and Cu was enriched at A, where it was intergranular. This indicates that at this time, the oxide film was also thinner. In Part B, the Ti and O contents were very high, and it was concluded there was a large amount of TiO_2_. From the analysis above, we conclude that the oxide films of AlTiNiCuCo_1.0_ alloy oxidized at 800 °C for 50 and 100 h were thinner.

**Figure 6:**
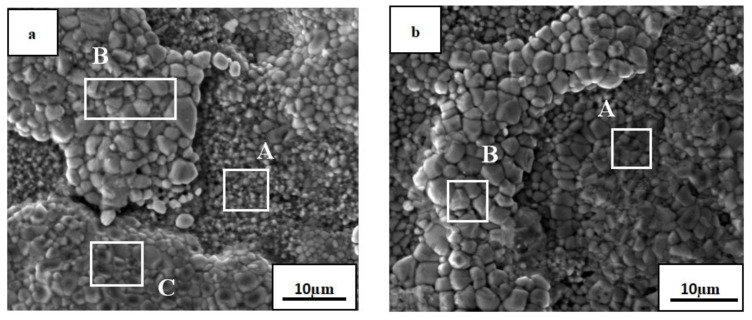
SEM micrographs of AlTiNiCuCo_1.5_ alloy after oxidation for (**a**) 50 h and (**b**) 100 h. A, B and C are selected areas for EDS analysis.

Figure 6 presents SEM micrographs of the oxide film of the AlTiNiCuCo_1.5_ alloy oxidized at 800 °C for 50 and 100 h, and Table 6 and Table 7 present the EDS analysis results of the corresponding regions in the two SEM images.

Compared with the microstructural morphology of the oxide films of AlTiNiCuCo_0.5_ and AlTiNiCuCo_1.0_ after 50 h oxidation, the microstructure distribution was very uneven and consisted of three obvious parts, namely, Part A with uniform and fine black spots, Part B consisting of large raised areas, and Part C consisting of small raised areas.

The EDS analysis indicated that Part A contained more Al, Co, and O. In XRD analysis, CoAl_2_O_4_ and Al_2_O_3_ were found in this part. The contents of O and Cu in Part B were more than 50%, which indicates that the Cu phase was enriched there. The contents of Ti, Co, and O in Part C were relatively high. We speculated that TiO_2_ and CoAl_2_O_4_ might be present in this small raised part. After 100 h oxidation, the microstructural morphology obviously consisted of two parts. In Part A, the particle size, which was uniform and fine after 50 h oxidation, became different, and there was also a large raised area in Part B. Compared with that after oxidation for 50 h, the Ti content in the Part A region was significantly increased. From this, combined with XRD analysis results, we presumed that TiO_2_ and CoAl_2_O_4_ were the two oxides in this region. The large raised area in Part B was basically the same as that after oxidation for 50 h, with high contents of Cu and O and low contents of other elements. There was still a solid solution rich in the Cu phase and possibly a small amount of CuO in Part B.

In summary, by comparing the microstructural morphology and EDS analysis results of AlTiNiCuCo_x_ (x = 0.5, 1.0, 1.5) series HEAs, we conclude the following: With the extension of the oxidation time, the grain size of each component of the alloys in the microstructure became larger, and the distribution of the microstructure became increasingly uneven, which indicates that the high-temperature oxidation resistance became progressively worse. The oxide films of the AlTiNiCuCo_1.0_ alloy oxidized for 50 and 100 h were thinner. At the same time, with the extension of oxidation time, the Ti content in the HEAs increased. From the XRD analysis results on the surface of the oxide film, it was found that other oxides composed of Ti and O were generated in addition to TiO_2_.

### 3.4. Cross-Sectional Morphology of The Oxide Film

**Figure 7:**
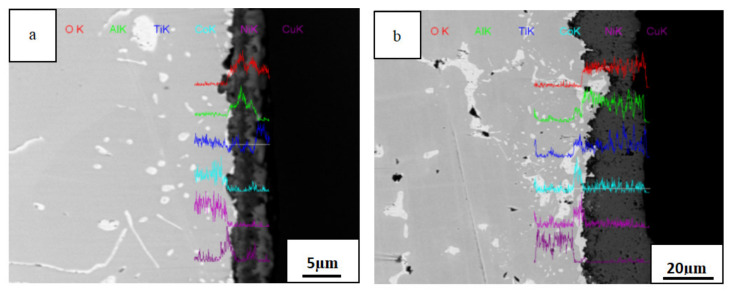
Line scanning energy spectra of oxidation layer sections of the AlTiNiCuCo_0.5_ alloy after oxidation for (**a**) 50 h and (**b**) 100 h.

Figure 7 presents line scanning energy spectrum diagrams of the cross section of the AlTiNiCuCo_0.5_ alloy oxide layer after oxidation for 50 and 100 h at 800 °C. It can be seen that after oxidation for 50 h, the oxide film was relatively continuous and uniform in thickness. When the oxidation time was 100 h, the thickness of the oxide film increased significantly, and the film morphology and thickness were uneven. In Figure 7, representing 50 h, it can be seen that there were high contents of Al, Ti, and O in the oxide layer. Combining this with the XRD analysis results of the oxide layer, it was concluded that the main components of the oxide layer were Al_2_O_3_ and TiO_2_. After oxidation for 100 h, the contents of Al and Ti in the oxide layer increased, and their distribution was more uniform. XRD analysis indicated that in addition to Al_2_O_3_ and TiO_2_, Ti_0.928_O_2_ and CoAl_2_O_4_ oxides also appeared.

**Figure 8:**
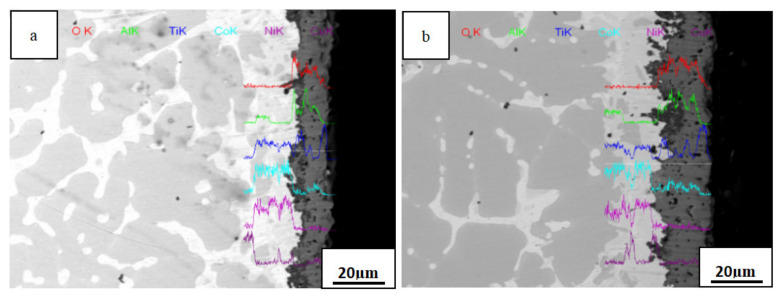
Line scanning energy spectra of oxidation layer sections of the AlTiNiCuCo_1.0_ alloy after oxidation for (**a**) 50 h and (**b**) 100 h.

Figure 8 presents line scanning energy spectrum diagrams of the cross section of the AlTiNiCuCo_1.0_ alloy oxide layer after oxidation for 50 and 100 h at 800 °C. After 100 h oxidation, the oxide film was thicker than that after 50 h, but the change in thickness was not apparent. This indicates that the oxide film of an HEA of this composition under high-temperature oxidation is relatively dense and plays a certain protective role for the alloy; thus, the alloy has better high-temperature oxidation resistance. It can be seen from Figure 8a, representing 50 h oxidation, that the oxide layer still contained large amounts of Al, Ti, and O elements. It was presumed that there were mainly two oxides, Al_2_O_3_ and TiO_2_, in the oxide layer. After oxidation for 100 h, the contents of Al and Ti in the oxide layer were greater, and the content of Co was significantly increased. Combining this with XRD analysis results, it was found that there were three oxides present: CoAl_2_O_4_, Al_2_O_3_, and TiO_2_.

**Figure 9:**
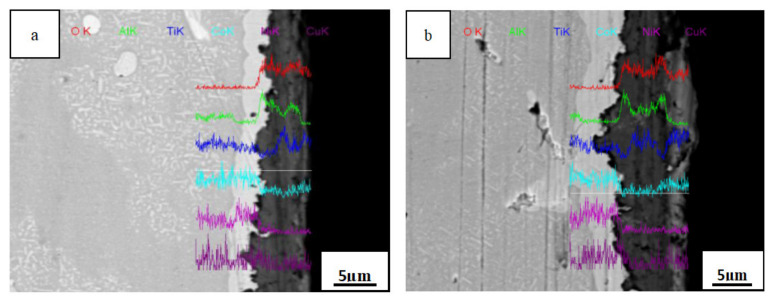
Line scanning energy spectra of oxidation layer sections of the AlTiNiCuCo_1.5_ alloy after oxidation for (**a**) 50 h and (**b**) 100 h.

Line scanning energy spectrum diagrams of the cross section of the AlTiNiCuCo_1.5_ alloy oxide layer after oxidation at 800 °C for 50 and 100 h are presented in Figure 9. In Figure 9, it can be seen that from the surface layer to the core, the sample was composed of an oxide layer, transition layer, and matrix. Combining this with the XRD phase analysis results, we concluded that for 50 h oxidation, three oxides—CoAl_2_O_4_, Al_2_O_3_, and TiO_2_—were produced in the oxide layer. The Co content in the transition layer increased significantly and CoAl_2_O_4_ was formed. With increasing oxidation time, the transition layer and oxide layer became thicker. Among them, the brighter part of the outer layer of the HEA appeared after 100 h oxidation, that is, the outer oxide layer, which contained more Cu and Ti. It was presumed that CuO and TiO_2_ were present in this part, and the surface was uneven. It was presumed that the peeling phenomenon of the outer oxide layer occurred due to the particle size of TiO_2_ in the outermost layer continuously growing.

By comparing the cross-sectional morphologies of oxide films across Figure 7, Figure 8 and Figure 9, we conclude the following: With the extension of the oxidation time, the oxides in the alloys increased. With increasing Co content, a transition layer gradually appeared. There was mainly Al_2_O_3_ and TiO_2_ in the oxide layer, and a small amount of CoAl_2_O_4_ was present. CoAl_2_O_4_ appeared in the transition layer.

### 3.5. Oxidation Mechanism

In the cross-sectional scanning images of AlTiNiCuCo_x_ (x = 0.5, 1.0, 1.5) series HEAs after oxidation for 50 and 100 h at 800 °C, an oxide layer was present in all samples. According to the analysis of Figure 7 and Figure 8, mainly the oxides Al_2_O_3_ and TiO_2_ were present in the oxide layer. This is because the oxidation activity sequence for the five constituent elements of the alloy in decreasing order of activity was Al, Ti, Co, Ni, and Cu. Among these, Al and Ti were highly active and were easily and preferentially oxidized. At the same time, according to the Equation (2)

(2) ΔG=ΔH−TΔS

where Δ*G* is Gibbs free energy, Δ*H* is the enthalpy changes, *T* is the temperature, Δ*S* is the Entropy change.

The free energy of the oxidation reaction of Al and Ti was lower than 0 and relatively low, so the reaction could proceed spontaneously, and stable Al_2_O_3_ and TiO_2_ were formed in the oxide layer. The oxidation layer was mainly composed of Al and Ti as alloy components that react with oxygen via direct contact. At this time, the interfacial reaction dominated.

In the oxidation layer cross section of the AlTiNiCuCo_1.5_ alloy after oxidation for 50 h and 100 h at 800 °C, we can see both a significant oxidation layer and transition layer. According to our analysis of Figure 9, the oxide CoAl_2_O_4_ was mainly present in the transition layer. This is due to the gradual outward diffusion of Co ions and Al ions meeting the gradual inward diffusion of O ions, whereupon CoAl_2_O_4_ oxide was formed in the transition layer. It can be seen that as the alloy was continuously oxidized, the oxide film continued to grow and gradually played an increasingly significant role, so the process transformed from the original interface reaction to the diffusion process, and diffusion became the dominant oxidation factor. This resulted in the appearance of one or more alloy oxides, mainly CoAl_2_O_4_.

Our analysis indicates that in the process of high-temperature oxidation, metal elements and oxygen in the alloy formed ions through direct contact reaction, and ions diffused and were transported in the oxide film, which is consistent with the high-temperature oxidation mechanism of NbCrMo_0.5_Ta_0.5_TiZralloy studied by Senkov et al. [16], US Air Force Laboratory. Al and Ti ions diffused out of the oxide film through the interface, and the distribution of Al and Ti ions gradually decreased from the oxide layer to the transition layer and to the matrix. Oxygen ions diffused through the oxide film to the substrate, and the concentration of oxygen ions gradually decreased from the oxide layer to the transition layer to the substrate. The concentration of Co ions increased gradually from the matrix to the transition layer. The distribution of Ni and Cu ions increased gradually from the transition layer to the matrix.

By analyzing the variation in the composition and concentration of various compounds in the oxide layer, transition layer, and matrix over time, we deduced the above oxidation mechanism, which is in excellent agreement with the actual observed results.

## 4. Conclusions

1. At 800 °C, AlTiNiCuCo_x_ (x = 0.5, 1.0, 1.5) series HEAs have certain high-temperature oxidation resistance and conform to the $\Delta m=K_{1}t^{n}$ (*n* = 1) oxidation mode.
  The oxidation resistance of the AlTiNiCuCo_x_ series HEAs at high temperature is closely related to the Co content.
2. With increasing oxidation time, the overall oxide content in the alloys increases, and the high-temperature oxidation resistance becomes progressively worse. With increasing Co content, a transition layer gradually appears. Among the alloys, the oxide films of AlTiNiCuCo_1.0_ oxidized for 50 and 100 h were thinner; AlTiNiCuCo_1.0_ has higher oxidation resistance at high temperature compared to alloys with other amounts of Co that were tested.
3. In the process of high-temperature oxidation of AlTiNiCuCo_x_ series HEAs, the interface reaction initially dominates, and metal elements and oxygen in the alloy react to form ions as a result of direct contact; then, ions diffuse and are transported in the oxide film, and the diffusion process gradually becomes the dominant oxidation factor. Because the initial position and diffusion direction differ for each ion, the distribution of concentrations in the oxide layer–transition layer–matrix eventually differs.

## Figures and Tables

**Table 1:** Oxidation weight increase per unit area of AlTiNiCuCo_x_ (x = 0.5, 1.0, 1.5) series HEAs at different oxidation times (g/m^2^).

Oxidation Time Co Content	25 h	50 h	75 h	100 h
x = 0.5	1.36	6.08	7.51	10.96
x = 1.0	0.93	1.70	3.83	5.03
x = 1.5	0.24	0.61	0.93	1.44

**Table 2:** EDS analysis results of the oxide film surface of AlTiNiCuCo_0.5_ alloy after oxidation for 50 h.

Element	O	Al	Ti	Co	Ni	Cu
wt%	32.29	06.51	54.26	01.60	01.45	03.90
at%	57.58	06.88	32.31	00.77	00.70	01.75

**Table 3:** EDS analysis results of the oxide film surface of AlTiNiCuCo_0.5_ alloy after oxidation for 100 h.

Element	O	Al	Ti	Co	Ni	Cu
wt%	30.46	03.90	58.12	02.18	01.44	03.90
at%	56.25	04.27	35.85	01.09	00.72	01.81

**Table 4:** EDS analysis results of the oxide film of AlTiNiCuCo_1.0_ alloy after oxidation for 50 h.

Element	O	Al	Ti	Co	Ni	Cu
wt%	28.49	11.32	41.12	06.18	03.68	09.22
at%	52.82	12.44	25.47	03.11	01.86	04.30

**Table 5:** EDS analysis results of the oxide film surface of AlTiNiCuCo_1.0_ alloy after oxidation for 100 h. Where A and B positions correspond to the marked areas in Figure 5.

Position	O	Al	Ti	Co	Ni	Cu
A	46.34	30.93	08.83	02.41	02.09	09.40
B	56.41	03.00	36.72	01.04	00.84	01.99

**Table 6:** EDS analysis results of the oxide film surface of AlTiNiCuCo_1.5_ alloy after oxidation for 50 h. Where A, B and C positions correspond to the marked areas in Figure 6.

Position	O	Al	Ti	Co	Ni	Cu
A	43.22	23.10	07.58	13.78	07.31	05.02
B	36.17	00.40	00.85	04.59	04.54	53.44
C	51.66	02.90	13.32	20.48	03.50	08.15

**Table 7:** EDS analysis results of the oxide film surface of AlTiNiCuCo_1.5_ alloy after oxidation for 100 h. Where A and B positions correspond to the marked areas in Figure 6.

Position	O	Al	Ti	Co	Ni	Cu
A	51.24	03.59	19.56	12.50	07.58	05.54
B	36.09	00.63	00.90	02.33	03.00	57.04

## Data Availability

All the data is available within the manuscript.
